# Comparative study of polymyxin B and colistin sulfate in the treatment of severe comorbid patients infected with CR-GNB

**DOI:** 10.1186/s12879-023-08339-0

**Published:** 2023-05-25

**Authors:** Jiale Wang, Binay Kumar Shah, Jian Zhao, Jie Xiong, Changhui Wang, Shuanshuan Xie

**Affiliations:** 1 Department of Respiratory Medicine, Shanghai Tenth People’s Hospital, Tongji University School of Medicine, Shanghai, 200072 China; 2 Tongji University School of Medicine, Shanghai, 200092 China; 3 Department of Emergency Medicine, Shanghai Tenth People‘S Hospital, Tongji University School of Medicine, Shanghai, 200072 China; 4 Department of Respiratory Medicine, ChongMing Branch of Shanghai Tenth People’s Hospital, Tongji University School of Medicine, Shanghai, 202157 China

**Keywords:** Colistin sulfate, Polymyxin B, CR-GNB, Clinical success, Microbial success

## Abstract

**Background:**

With the difficulties in choosing colistin sulfate and polymyxin B sulfate (PBS) for carbapenem-resistant gram-negative bacteria (CR-GNB), we compared the efficacy and safety of these two old polymyxins in treatment of critically ill patients infected with CR-GNB infection.

**Methods:**

One hundred four patients infected with CR-GNB in ICU were retrospectively grouped by PBS (68 patients) or colistin sulfate (36 patients). Clinical efficacy including symptoms, inflammatory parameters, defervescence, prognosis and microbial efficacy were analyzed. Hepatotoxicity, nephrotoxicity, and hematotoxicity were evaluated by TBiL, ALT, AST, creatinine, and thrombocytes.

**Results:**

Demographic characteristics between colistin sulfate and PBS were not significantly different. Most of the CR-GNB were cultured in respiratory tract (91.7% vs 86.8%), and almost all were polymyxin-sensitive (98.2% vs 100%, MIC ≤ 2 μg/ml). The microbial efficacy in colistin sulfate (57.1%) was significantly higher than PBS (30.8%) (*p* = 0.022), however, no significant difference in clinical success was seen in both groups (33.8% vs 41.7%), as well as mortality, defervescence, imaging remission, days in the hospital, microbial reinfections, and prognosis, and almost all patients defervesce within 7 days (95.6% vs 89.5%).

**Conclusions:**

Both polymyxins can be administrated in critically ill patients infected with CR-GNB and colistin sulfate is superior to PBS in microbial clearance. These results highlight the necessity of identifying CR-GNB patients who may benefit from polymyxin and who are at higher risk of mortality.

## Introduction

The effectiveness of antibiotics, which have revolutionized medicine and saved millions of lives, is in a precarious position due to the rapid growth of resistant infections brought on by multidrug resistance gram-negative bacteria (MDR-GNB), and death rate of 47% or higher, longer stays in the intensive care unit (ICU) or in hospital have all been observed with CR-GNB infections [1]. MDR-GNB especially Carbapenem-resistant* Klebsiella pneumonia* (CRKP), Carbapenem-resistant* Acinetobacter baumannii* (CRAB), Carbapenem-resistant* Pseudomonas aeruginosa* (CRPA), and Carbapenem-resistant *Escherichia coli* (CRECO), have been labeled a critical threat by the WHO [2]. The all-cause mortality rate of CRKP was reported to be 34.1% to 52.8% [3]. According to the 2022 CHINET China Bacterial Resistance Monitoring Network statistics, CRKP surged from 2.9% in 2005 to 24.2% in 2022 for resistance of imipenem. CRPA and CRAB were as high as 22.1% and 71.2% respectively [4]. Alternative strategies are needed to battle this infection.

CR-GNB infection therapy options that use novel antibiotics are limited. Polymyxins are bactericidal drugs that encompass a variety of chemicals. They were generated from *Paenibacillus polymyxa* and were clinically useful in the 1950s [5]. However, because of their toxic effects and the accessibility of those other safer and more efficient antibacterial medications, their uses have declined significantly since the 1970s [6]. With the present rapid growth of CR-GNB and advancements in polymyxin preparation technology, an old antibiotic has been reintroduced.

In addition to neutralizing lipopolysaccharides (LPS), polymyxin binds to LPS and phospholipids in the outer cell membrane of GNB. This competitively displace divalent cations from the phosphate groups of membrane lipids cause disruption of the outer cell membrane, leakage of intracellular contents, and bacterial death [7]. Three different forms of polymyxins are currently available on domestic and international markets: colistimethate sodium (CMS), polymyxin B sulfate (PBS), and colistin sulfate. Nephrotoxicity and neurotoxicity are the most frequent side effects of polymyxin [8]. Nearly 60% to 70% of CMS is eliminated by the kidneys which are an unactivated pre-drug that must be converted into active polymyxin in vivo to have bactericidal effects. The latter two medications can operate directly and do not need to be converted; they are primarily excreted through non-renal pathways [9]. On CMS and PBS, various clinical trials have been carried out but which drug should be used more frequently to treat infections brought on by CR-GNB in order to ensure higher efficacy and lower incidence of side effects has not yet been determined. CMS was found to be less nephrotoxic than PBS in preliminary research [10]. As CMS has to be covered to polymyxin in vivo before it can exert its bactericidal action, this delays the onset of its activities at a time when it’s critical to treat persons with serious infections as soon as possible [11]. As a result, clinicians prefer PBS without conversion. Even more recently, numerous studies have demonstrated that PBS has a lower risk of acute kidney damage [12]. Indeed, nephrotoxicity and other adverse effects are only transient and reversible when the dosage is reduced or the medication is discontinued. Colistin sulfate is being used in China since 2018 [13]. Colistin sulfate and PBS have comparable metabolic pathways and can both function without conversion. Extravascular distribution and penetration into the outer tissues of PBS are little known, although it is hypothesized that it is comparable to that of colistin sulfate, with generally low penetration into the lungs, pleura, bones, and central nervous system [14].

Here we conducted a retrospective study on the efficacy of PBS and colistin sulfate in the treatment of CR-GNB in critically ill patients to find a reference for the clinical selection of these two drugs.

## Materials and methods

### Ethics

The study was approved by the Ethics committee of the Shanghai Tenth People’s Hospital. The need for written informed consent was waived by the Ethics committee of the Shanghai Tenth People’s Hospital due to retrospective nature of the study, and the study was carried out in accordance with relevant guidelines and regulations.

### Study design and patients selection

The inclusion criteria were (I) administration of PBS and colistin sulfate (II) age > 18 years old; (III) diagnosed with pneumonia, bacteremia, urinary tract infection or acute suppurative peritonitis or meningitis; (IV) CR-GNB in sputum, blood, mid-stream urine, cerebrospinal fluid, abdominal drainage or bronchial alveolar lavage fluid were cultured or observed by microscopy before polymyxin administration.

The exclusion criteria were: (I) patients received polymyxin for less than 3 days or died within 2 days; (II) infected by multidrug-resistant gram-positive cocci during administration; (III) a combination of PBS and colistin sulfate. (IV) only infected by bacteria intrinsically resistant to polymyxin.

We conducted the retrospective study in Shanghai Tenth people hospital, China from November 1, 2021 to November 30, 2022. All the patients were given polymyxin in combination of other antibiotics. The therapeutic polymyxins included PBS (Unified drug code: XN0000030084471, produced by Shanghai No.1 Biochemical & Pharmaceutical Co., Ltd) and colistin sulfate (Unified drug code: XN0000030092970, produced by New Asiatic Pharmaceutical Co., Ltd).

### Clinical data collection

Patients' demographic characteristics (age, sex, comorbidities, mechanical ventilation or not), infection status (infection site, pathogenic bacteria, polymyxin MIC distribution), therapeutic regimens (treatment duration, combination of other regiments), laboratory data (leukocyte count, percent of neutrophilic granulocyte, C-reactive protein and amyloid protein), radiograph, temperature, length of hospital stay and prognosis were collected.

### Outcomes

The primary outcome included clinical success and microbiological success, where clinical success was defined as the disappearance of clinical signs and symptoms, normalization of at least two inflammatory markers (including leukocyte count, percent of neutrophilic granulocyte, C-reactive protein and amyloid protein) and resolution of fever within 7 days of treatment, whereas clinical failure was defined as no improvement in signs and symptoms, less than two remissions or significant progression of inflammatory markers, or death of the patient within 7 days of polymyxin therapy. Microbiological success was defined as the clearance of the CR-GNB in sputum, blood, mid-stream urine, cerebrospinal fluid, drainage fluid or lavage fluid on two consecutive cultures after 7 days of treatment. The secondary outcome included the proportion of normalized inflammatory parameters and the variation in those who did not return to normal, the proportion of defervescence and the days it took, the proportion of imaging remission, microbial reinfections, the days in the hospital, and 14-day, 28-day and total mortality.

Among the outcomes, normalization of inflammatory markers was defined as leukocyte count < 10*10^9^/L, neutrophilic granulocyte < 75%, C-reactive protein < 8.2 mg/L and amyloid protein < 10 mg/L, and the variation for those who did not return to normal was defined as the maximum of the difference between 2 days pre-dose and 7 days post-dose. The microbial reinfections were defined as additional CR-GNB observed by the culture of sputum, blood, mid-stream urine, cerebrospinal fluid or drainage lavage fluid within 7–14 days after dosing. The days in the hospital counted from the initiation of polymyxin administration and the prognosis is mainly determined by 14 days, 28 days and total mortality.

### Safety assessment

The study compared the hepatotoxicity, nephrotoxicity and hematotoxicity between PBS and colistin sulfate. The hepatotoxicity was evaluated by the abnormality of TBiL(total bilirubin), ALT(alanine aminotransferase) or AST(aspartate aminotransferase), and the twice of baseline or the abnormality of serum creatinine was used to indicate nephrotoxicity. The hematotoxicity was assessed by the decrease of thrombocytes. Patients who had abnormal laboratory results as described above before polymyxin administration were excluded, and the data on safety assessment mentioned above were collected within 24 h before discontinuation of the polymyxin.

### Statistical analyses

Statistical analyses were performed by R 4.2.1. Categorical variables were expressed as percentages, and continuous variables were expressed as mean ± standard deviation if normal distribution or median (Min, Max) if skewed distribution. The variable distribution was assessed by the Shapiro test. The Chi-square or Fisher’s exact test (two-tailed, expectation < 5) was used to compare categorical variables, while the unpaired Student’s t-test or non-parametric Mann–Whitney test was used to compare continuous variables. The Kaplan–Meier method was used for survival analysis and the log-rank test was used for comparison between groups. *P* value < 0.05 was considered statistically significant.

## Results

### Selection of patients

**Fig. 1:**
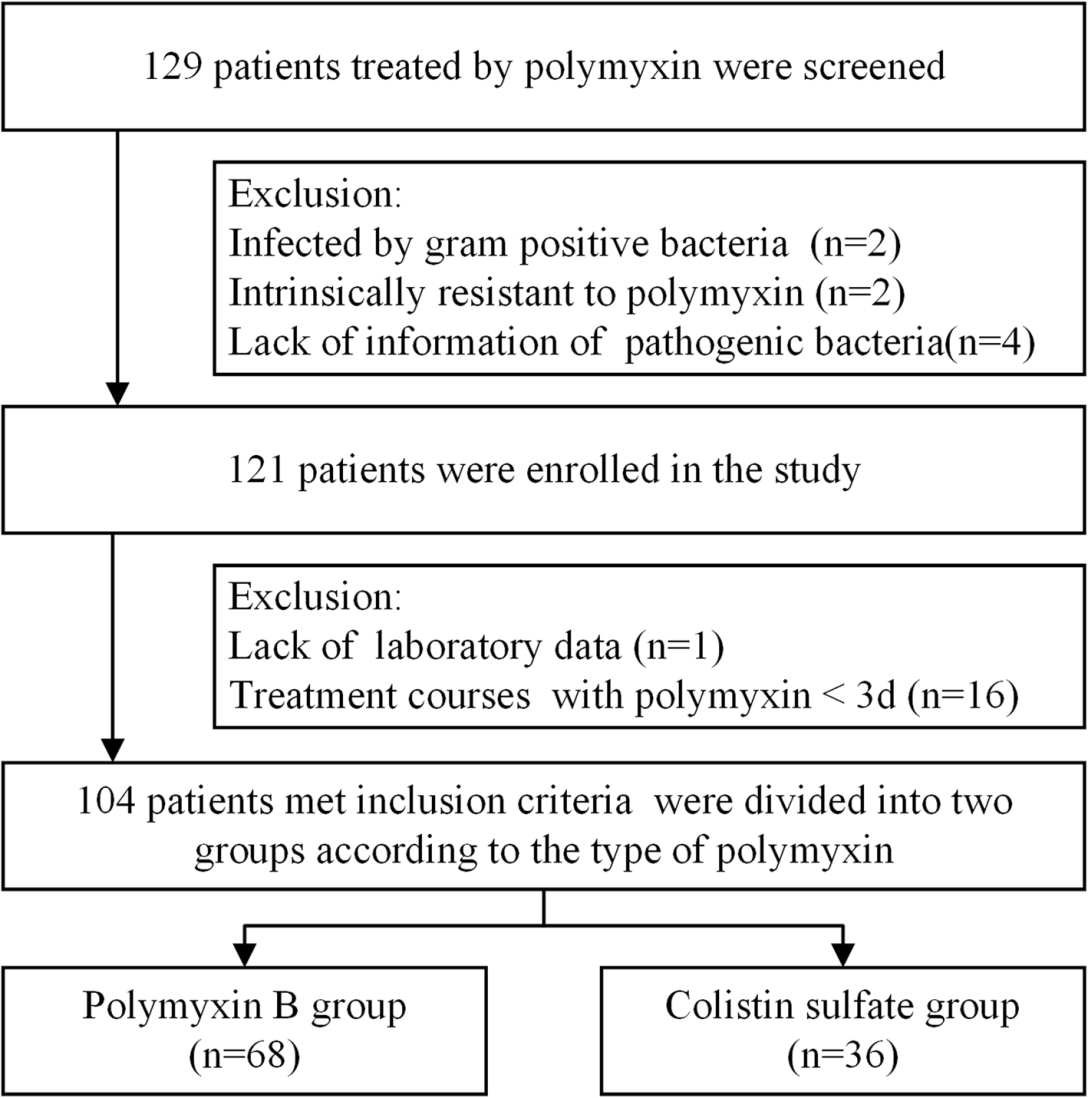
Study population flow chart

A total of 129 patients were screened in this study and 121 patients infected with CR-GNB and treated with polymyxin were retained after 8 patients were excluded, including 1 patient infected by methicillin-resistant Staphylococcus aureus (MRSA), 1 patient infected by only *Enterococcus faecium,* 2 patients infected by only *Proteus mirabilis* or *Serratia marcescens* and 4 patients with missing microbiological information. Due to lack of laboratory information of 1 patient and 16 patients treated with polymyxin for less than 3 days were further excluded, finally, 104 patients were enrolled, all in ICU and were divided into two groups based on the type of polymyxin: PBS and colistin sulfate groups (Fig. 1).

### Characteristics of patients

All of the patients were admitted to the ICU. 104 patients infected by CR-GNB and administrated by polymyxin were enrolled in the study and divided into two groups: PBS and colistin sulfate group.

As Table 1 presented, there was no significant difference between the two groups in terms of demographic characteristics. Both were predominantly older men, and nearly one-third of the participants had comorbidity with diabetes mellitus (DM) (38.2% vs 38.9%), chronic kidney disease (CKD) (33.8% vs 33.3%) and sepsis (32.4% vs 27.8%), and nearly half had respiratory comorbidities (47.1% vs 55.6%). The highest number of people had a combination with cardiovascular disease (CVD) (70.6% vs 75.0%) and the lowest with malignant tumors (13.2% vs 2.8%) and immunosuppressants (0% vs 5.6%). Mechanical ventilation was administered to almost all patients (91.2% vs 97.2%) during treatment and was predominantly invasive because of their admission to the ICU.

**Table 1:** Characteristics of patients of polymyxin B and colistin sulfate groups

	**Polymyxin B (*N* = 68)**	**Colistin sulfate (*N* = 36)**	***P*.value**
**Demographic characteristics**			
Age Median (Min, Max)	70.00 (23.00, 89.00)	71.00 (19.00, 97.00)	0.505
Sex(Male)	52 (76.5%)	26 (72.2%)	0.634
Comorbidities			
Diabetes Mellitus	26 (38.2%)	14 (38.9%)	0.948
Chronic Liver Diseases	14 (20.6%)	5 (13.9%)	0.400
Chronic Kidney Diseases	23 (33.8%)	12 (33.3%)	0.960
Cardiovascular Disease	48 (70.6%)	27 (75.0%)	0.633
Respiratory Comorbidities	32 (47.1%)	20 (55.6%)	0.410
Sepsis	22 (32.4%)	10 (27.8%)	0.631
Malignant Tumor	9 (13.2%)	1 (2.8%)	0.159
Immunosuppressants	0 (0%)	2 (5.6%)	0.118
Mechanical ventilation			0.434
Invasive	56 (82.4%)	30 (83.3%)	
Non-invasive	6 (8.8%)	5 (13.9%)	
No mechanical ventilation	6 (8.8%)	1 (2.8%)	
**Infection status**			
Infection site			1.000
Pneumonia	40 (58.8%)	21 (58.3%)	
Blood	1 (1.5%)	0 (0%)	
Abdomen	3 (4.4%)	1 (2.8%)	
Urinary tract	3 (4.4%)	2 (5.6%)	
≥ two infection sites	21 (30.9%)	12 (33.3%)	
Pathogenic bacteria			0.478
CRAB^a^	19 (27.9%)	14 (38.9%)	
CRPA^b^	8 (11.8%)	4 (11.1%)	
CRKP^c^	25 (36.8%)	9 (25.0%)	
Others^d^	3 (4.4%)	0 (0%)	
≥ two species of bacteria	13 (19.1%)	9 (25.0%)	
Polymyxins MIC distribution^e^	*n* = 55	*n* = 33	0.775
MIC = 0.5 μg/ml	50 (90.9%)	29 (87.9%)	
MIC = 1 μg/ml	0 (0%)	1 (3.0%)	
MIC = 2 μg/ml	4 (7.3%)	3 (9.1%)	
MIC = 4 μg/ml	1 (1.8%)	0 (0%)	
**Therapeutic regimens**			
Treatment duration (d) Median (Min, Max)	9.00 (3.00, 32.00)	11.00(3.00, 25.00)	0.123
Administration			0.275
Intravenous drip	45 (66.2%)	20 (55.6%)	
Inhalation	5 (7.4%)	1 (2.8%)	
Intravenous and Inhalation	18 (26.5%)	15 (41.7%)	
Combination			0.175
Meropenem or Imipenem	28 (41.2%)	9 (25.0%)	
Tigecycline	15 (22.1%)	13 (36.1%)	
Cefoperazone/Sulbactam or Piperacillin/Tazobactam	25 (36.8%)	14 (38.9%)	

**Fig. 2:**
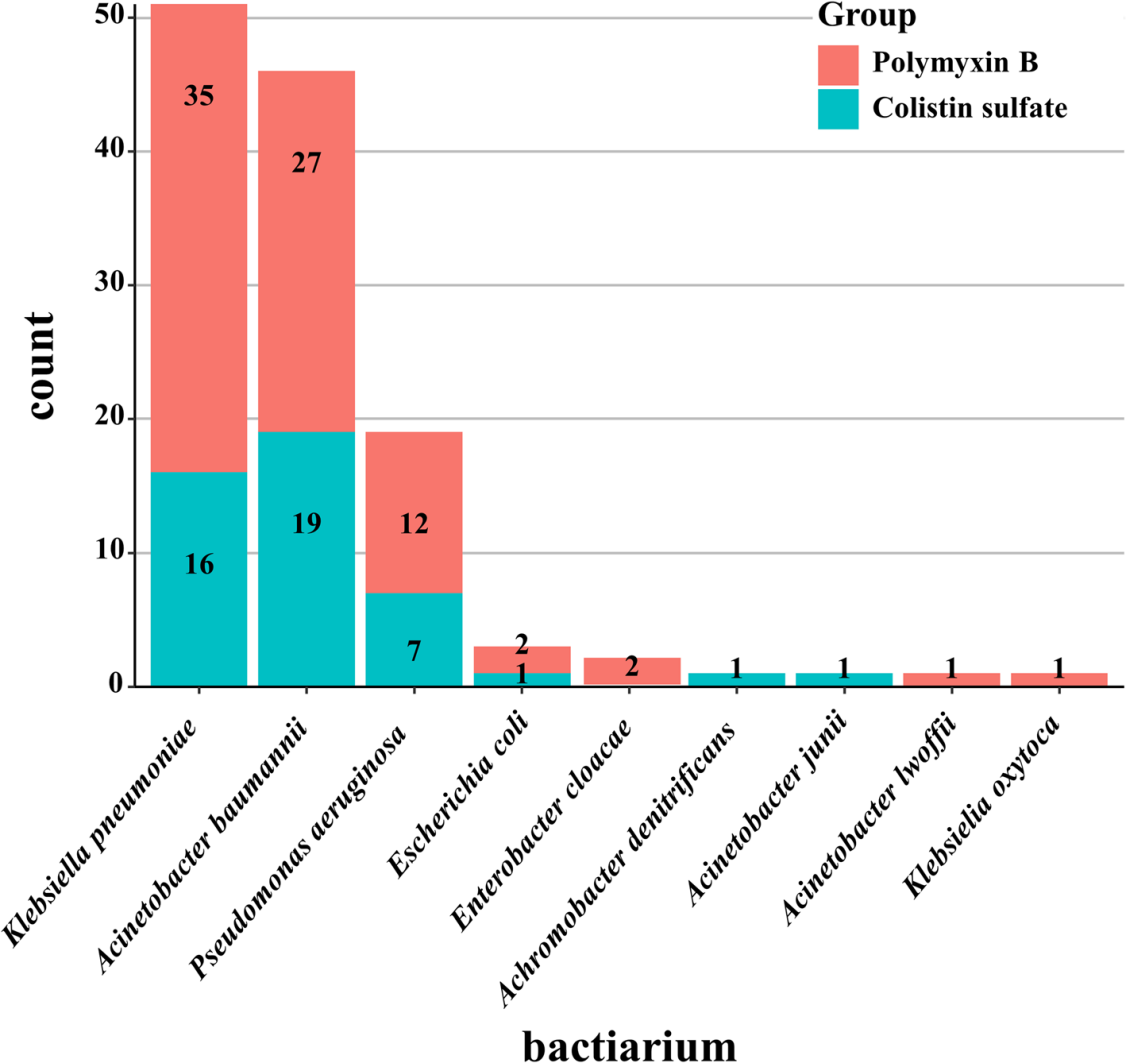
The distribution of CR-GNB isolated from patients in the present study. The number of patients infected by the pathogenic bacterium is marked in the center of the column

As for infection status, there was no significant difference observed. Most of the pathogenic bacterium were cultured in sputum or bronchoalveolar lavage fluid (86.8% vs 91.7%), 18 in blood (17.6% vs 16.7%), 11 in mid-stream urine (13.2% vs 5.6%) and 4 in abdominal drainage (4.4% vs 2.8%) respectively when patients with more than 2 infection sites were counted together. The pathogenic bacteria were predominantly CRAB(27.9% vs 38.9%), CRPA(11.8% vs 11.1%) and CRKP(36.8% vs 25.0%), almost all polymyxin-sensitive according to European Committee for Antimicrobial Susceptibility Testing (EUCAST) criteria (98.2% vs 100%, MIC ≤ 2 μg/ml). Approximately one-quarter of the patients were infected by at least 2 CR-GNB (19.1% vs 25.0%), and when they were included, the distribution of CR-GNB isolated from patients was as shown in Fig. 2, with CRKP accounting for the most at 40.8%, followed by CRAB at 36.8% and CRPA at 15.2%, in addition to other CR-GNB such as *Escherichia coli*, *Enterobacter cloacae*, *Acinetobacter junii* etc. also accounted for a certain percentage.

No statistically significant differences were observed in terms of therapeutic regimens either. All of the participants pretreated or combined with other antibiotics. In the PBS group, 28 (41.2%) were combined with meropenem or imipenem, 15 (22.1%) with tigecycline, 25 (36.8%) with cefoperazone/sulbactam or piperacillin/tazobactam. Similar in the colistin sulfate group, 9 (25.0%) were combined with meropenem or imipenem, 13 (36.1%) with tigecycline, and 14 (38.9%) with cefoperazone/sulbactam or piperacillin/tazobactam. The median duration between them was around 10 days (9d vs 11d), but in terms of administration, the PBS group was dominated by 45 (66.2%) people on intravenous drip, 18 (26.5%) on intravenous drip combined with inhalation and 5 (7.4%) on inhalation alone. In the colistin sulfate group, 20 (55.6%) were treated by intravenous drip, 15 (41.7%) by intravenous drip combined with inhalation and only 1 (2.8%) by inhalation alone.

### Therapeutic efficacy

**Fig. 3:**
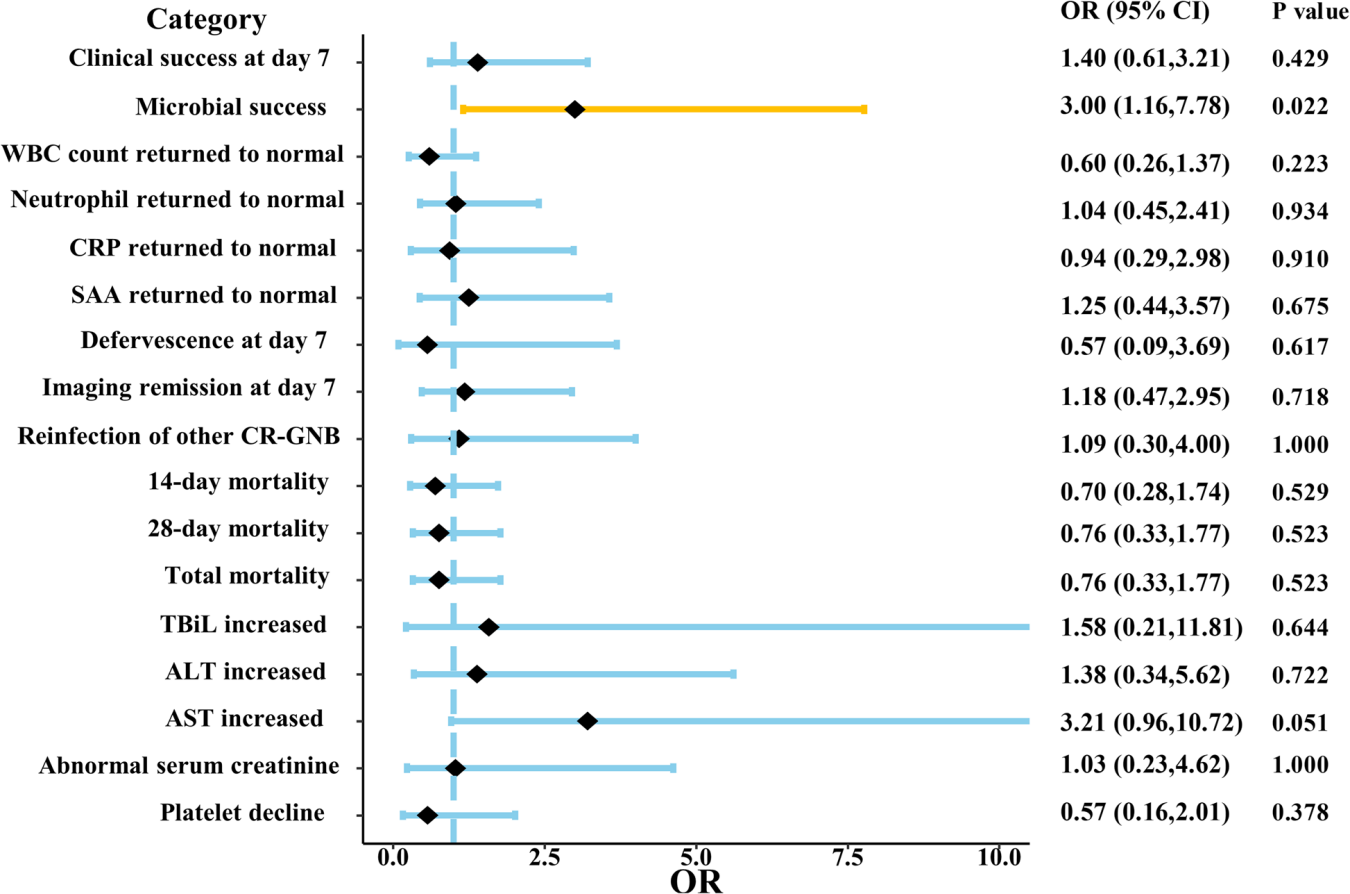
The forest of outcomes and safety between polymyxin B and colistin sulfate group. OR (odds ratio) was calculated with the control of polymyxin B group. Colistin sulfate caused a higher microbial clearance rate than B significantly (30.8% vs 56.7%, *p* = 0.021)

In terms of primary outcomes, as shown in Table 2 (or Fig. 3), microbial clearance was successful in 16 (30.8%) and failed in 36 (69.2%) in the PBS group, compared to 16 (57.1%) and 12 (42.9%) in the colistin sulfate group, and colistin sulfate caused a higher microbial clearance rate than PBS significantly (30.8% vs 57.1%, *p* = 0.022). However, there was no significant difference in clinical success between PBS and colistin sulfate groups.

**Table 2:** Comparison of therapeutic efficacy between polymyxin B and colistin sulfate groups

	**Polymyxin B (*N* = 68)**	**Colistin sulfate (*N* = 36)**	***P*.value**
**Primary Outcome**			
Clinical success on day 7	23 (33.8%)	15 (41.7%)	0.429
Microbial success	16 (30.8%),*n* = 52	16 (57.1%),*n* = 28	0.022
**Secondary Outcome**			
Inflammation index on day 7			
WBC^a^ count returned to normal	46 (67.6%)	20 (55.6%)	0.223
Neutrophils returned to normal	24 (35.3%)	13 (36.1%)	0.934
CRP^b^ returned to normal	10 (14.7%)	5 (13.9%)	0.910
SAA^c^ returned to normal	11 (16.2%)	7 (19.4%)	0.675
Variation of WBC(*109/L) Median (Min, Max)	5.02 (-15.00, 20.00),*n* = 22	4.02 (-0.790, 50.6),*n* = 16	0.827
Variation of neutrophil (%)Mean (± SD)	5.45 (± 5.89),*n* = 44	6.30 (± 4.63),*n* = 23	0.518
Variation of CRP (mg/L) Median (Min, Max)	52.80 (-30.40, 197.00),*n* = 58	67.1 (2.90, 195),*n* = 31	0.399
Variation of SAA (mg/L) Median (Min, Max)	90.70(-341.00, 509.00),*n* = 57	28.3 (-471, 533),*n* = 29	0.369
Defervescence at day 7	45(95.6%),*n* = 48	17 (89.5%),*n* = 19	0.617
Time to defervescence (d) Median (Min, Max)	2.00 (1.00, 7.00),*n* = 45	2.00 (1.00, 3.00),*n* = 17	0.212
Imaging remission on day 7	26 (51.0%),*n* = 51	16(55.2%),n = 29	0.718
Reinfection of other CR-GNB^d^	7 (10.3%)	4 (11.1%)	1.000
Length of hospital stay (d) Median (Min, Max)	13.00(3.00, 61.00)	14.0 (3.00, 44.0)	0.905
14-day mortality	21 (30.9%)	9 (25.0%)	0.529
28-day mortality	27 (39.7%)	12 (33.3%)	0.523
Total mortality	27 (39.7%)	12 (33.3%)	0.523

^a^WBC: white blood cell

^b^CRP: C-reactive protein

^c^SAA: serum amyloid A

^d^CR-GNB: carbapenem-resistant gram-negative bacteria

Moreover, no significant differences were observed in terms of inflammatory markers, either in the number of patients who returned to normal or in the variation of patients who did not return to normal. Although more than half of the patients could normalize their white blood cell counts(WBC) (67.6% vs 55.6%) within 7 days, only about 1/3 of the patients had a normal neutrophil (35.3% vs 36.1%), and even fewer had a normal C-reactive protein (CRP) (14.7% vs 13.9%) and serum amyloid A(SAA) (16.2% vs 19.4%). Additionally, no significant differences were observed in defervescence, imaging remission, days in the hospital, or microbial reinfections. Almost all patients successfully achieved defervescence within 7 days (95.6% vs 89.5%), but the imaging remission rate was only about half (51.0% vs 55.2%). Moreover, in the PBS and colistin sulfate group, 7 (10.3%) and 4 (11.1%), respectively, were reinfected with other CR-GNB during the dosing of polymyxin.

**Fig. 4:**
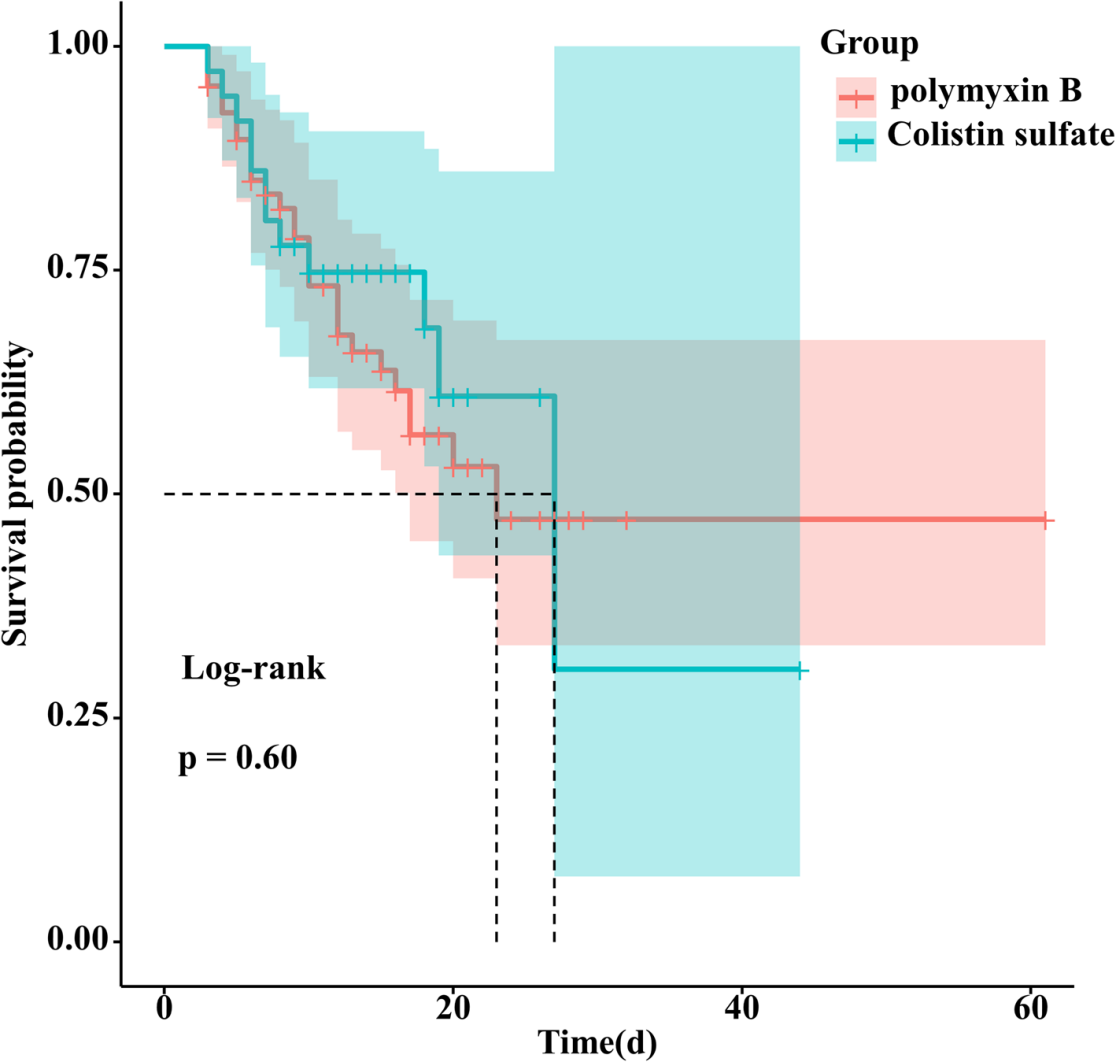
The survival curves and 95% confidence interval of polymyxin B and colistin sulfate group. 28-day mortality rate was 32.4% and 30.0% in patients with polymyxin B and colistin sulfate, respectively (log-rank, P = 0.61)

No statistical difference was observed in prognostic outcomes (14 days, 28 days, and total mortality). The total mortality for PBS and colistin sulfate groups were 39.7% and 33.3%, respectively. Survival analysis (Fig. 4) also showed that the median survival time was 23 days in the PBS group and 27 days in the colistin sulfate and that there was no statistical difference in survival probability between the two groups. However, the survival curves in both groups gradually became flatter as the duration of administration, suggesting that polymyxin is effective and can reduce the risk of death resulted from CR-GNB infection.

### Safety evaluation

As Table 3 (or Fig. 3) presented, both PBS and colistin sulfate can cause hepatotoxicity, predominantly AST elevation, nephrotoxicity, and thrombocytopenia, but no patients withdrew polymyxin due to those side effects. The side effects of PBS were mainly thrombocytopenia, with 10 (21.2%) patients suffering thrombocytopenia within 7 days of the administration, followed by nephrotoxicity (14.6%), elevated AST (10.9%), ALT (10.0%) and elevated TBiL in only 2 (3.9%) patients. In the colistin sulfate group, the side effects were predominantly AST elevation, with 9 (28.1%) patients suffering AST elevation during treatment, followed by nephrotoxicity (15.0%), ALT elevation (13.3%), thrombocytopenia in 4 patients (13.3%) and TBiL elevation in the least (6.1%). However, there was no significant difference in safety evaluation between PBS and colistin sulfate groups.

**Table 3:** Comparison of safety evaluation between polymyxin B and colistin sulfate groups

	**Polymyxin B (*N* = 68)**	**Colistin sulfate (*N* = 36)**	***P*.value**
**Hepatotoxicity**			
Total bilirubin increased	2 (3.9%),*n* = 51	2 (6.1%),*n* = 33	0.644
Alanine aminotransferase increased	5 (10.0%),*n* = 50	4 (13.3%),*n* = 30	0.722
Aspartate aminotransferase increased	5 (10.9%),*n* = 46	9 (28.1%),*n* = 32	0.051
**Nephrotoxicity**			
Abnormal serum creatinine	6 (14.6%),*n* = 41	3 (15.0%),*n* = 20	1.000
**Hematotoxicity**			
Platelet decline	10 (21.2%),*n* = 47	4 (13.3%),*n* = 30	0.378

## Discussion

The emergence of CR-GNB becomes a global public health concern and studies have shown that receiving inappropriate antibiotics and delaying the start of early and first acceptable antimicrobial therapy are potential risk factors for death rates and protracted organ dysfunction. The mortality of patients receiving inappropriate antibiotic therapy ranged from 45 to 63% [15]. The primary issue with treating CR-GNB is still selecting the appropriate medications and overcoming their difficulties. The purpose of this study was to compare the efficacy and safety of both old polymyxins in the treatment of critically ill patients with CR-GNB infections.

In this study, 104 patients infected by CR-GNB were included and were divided into two groups and given PBS and colistin sulfate in a combination with other antibiotics. All the patients were in ICU and almost on mechanical ventilation predominantly invasive (91.2% vs 97.2%) and had various other comorbidities predominantly CVD followed by respiratory, DM, CKD, sepsis, etc. The predominant pathogenic bacteria were CRKP followed by CRAB and CRPA with other certain pathogens. The primary outcome which was a clinical and microbial success after 7 days of treatment shows a significant microbial clearance rate of colistin sulfate than PBS (57.1% vs. 30.8%) whereas there is no significant difference in clinical success between the two groups (41.7% vs. 33.8%) respectively.

These findings are encouraging when compared to the outcomes of studies where patients were treated with PBS and colistin sulfate for CR-GNB infections. A retrospective multicenter study in patients with CR-GNB infection who were treated with PBS, mostly in combination with other anti-CR-GNB antibiotics to which all strains remained sensitive, found a bacterial eradication rate of 77.65% with 28 days mortality of 40% concluded timely and appropriate use of PBS may have a positive impact on clinical outcomes in the treatment of CR-GNB infections [1]. Another study that uses colistin sulfate monotherapy and its combination with other antimicrobials in the treatment of CR-GNB in both ICU and non-ICU patients shows clinical efficacy (94.4% vs. 73.1%) and microbial clearance rate (74.1% vs. 50.0%) were significantly higher in combination therapy with 28 days mortality rate of 5.6% vs. 11.5% in combined vs monotherapy [13].

A systemic review shows mortality ranged from 8 to 56% in colistin sulfate-treated patients and from 31 to 61% in PBS-treated patients and there was no significant difference in unadjusted mortality between patients treated with colistin sulfate and PBS (RR = 0.71, 95% CI 0.45–1.13; I^2^ = 80%) [16].

In contrast to our study which included severe co-morbid patients of ICU, the 28 days mortality which is a secondary outcome shows there was no significant difference in PBS (39.7%) and colistin sulfate (33.3%) groups. Also there was no statistical difference in survival probability between PBS (23 days) and colistin sulfate (27 days) groups and survival curves in both groups gradually become flatter as the duration of administration, suggesting that both polymyxins are effective and can reduce the risk of death resulting from CR-GNB infections. There were no significant differences between the two groups in terms of age, underlying condition, risk factors towards CR-GNB infection, kind of infection, pathogenic distribution, the period from positive culture to initiation of anti-infective treatment, or treatment duration. Although more than half of the patients normalized their WBC counts within 7 days, only about 1/3 of patients had normal neutrophils with fewer with normal CRP and serum amyloid. Almost all patients successfully achieve defervescence and the imaging remission rate was only about half (51.0% vs. 55.2%).

Being elderly, high daily doses, having underlying diseases such as DM and use of concomitant nephrotoxic drugs were independent predictors of polymyxin-induced nephrotoxicity, and the majority of events were reversible [17]. A systemic review shows the pooled incidence of nephrotoxicity caused by colistin sulfate was 48%, while that of PBS (38%) induced nephrotoxicity was 10% lower than colistin sulfate [17].

In our study, there was no significant difference in safety evaluation. 14.6% of patients in the PBS and 15.0% of patients in the colistin sulfate group showed nephrotoxicity. Other toxicities between both groups included thrombocytopenia (21.2% vs 13.3%), increased AST (10.9% vs 28.1%), ALT (10.0% vs 13.3%), and TBiL (3.9% vs 6.1%). However, none of the patients withdrew the polymyxin because of these side effects. Therefore, both polymyxins were safe enough to ensure their administration in CR-GNB infections. Moreover, in the PBS and colistin sulfate groups, 7 (10.3%) and 4 (11.1%) patients, respectively, were reinfected with other CR-GNB during the dosing of polymyxin.

There are some limitations of our study. A retrospective, single-center study involving 104 patients impacted the generalizability of our findings. During treatment, based on guidelines and personal experience physicians made decisions regarding dosage, administration, and duration without therapeutic drug monitoring, and without monitoring the plasma and tissue concentrations of both polymyxins. Individual physicians decided to combine polymyxin with other antimicrobial drugs, which create an introduction bias. In addition, it is challenging to distinguish between adverse reactions and the efficacy of polymyxin alone, which may contribute certain limitations in the results. The majority of patients in this study were in invasive mechanical breathing combined with sedative drugs, thus it is highly possible that neurotoxic symptoms might be overlooked, making it harder to detect neurotoxicity in those individuals. Some cases of microbial invasion might have been managed as clinical infections, and the concomitant administration of other antibiotics could not be evaluated.

Furthermore, our finding establishes a foundation for selecting combination regimens against CR-GNB infections. Also, our study encourages further research on this topic, especially those evaluating supplementary variables like concomitant infections, other medications, and treatment-related complications.

## Conclusions

In summary, our study shows both polymyxins can be administrated in critically ill patients infected with CR-GNB and colistin sulfate is superior to PBS in microbial clearance, but there was no significant difference in clinical efficiency between PBS and colistin sulfate. These results underscore the need to more quickly identify patients with CR-GNB who may benefit from the judicious use of polymyxin and who are at higher risk of mortality. Furthermore, our finding suggests the use of combination regimens against CR-GNB infections. Also, our study encourages further research on this topic, especially the combination with colistin sulfate, for those evaluating supplementary variables like concomitant infections, other medications, and treatment-related complications in large sample sizes, and multicenter studies to find precise strategies to optimize the efficacy and safety of these drugs.

## Data Availability

The datasets used and/or analyzed during the current study are available from the corresponding author on reasonable request.

## Abbreviations

CKD: Chronic kidney disease
CMS: Colistimethate sodium
CRAB: Carbapenem-resistant *Acinetobacter baumannii*
CRECO: Carbapenem-resistant* Escherichia coli*
CR-GNB: Carbapenem-resistant* Gram-negative bacteria*
CRKP: Carbapenem-resistant* Klebsiella pneumonia*
CRP: C-reactive protein
CRPA: Carbapenem-resistant* Pseudomonas aeruginosa*
CVD: Cardiovascular disease
DM: Diabetes mellitus
ICU: Intensive care unit
LPS: Lipopolysaccharides
MDR-GNB: Multidrug resistance gram-negative bacteria
MIC: Minimal inhibitory concentration
PBS: Polymyxin B sulfate
SAA: Serum amyloid A
WBC: White blood cell

## References

[1] Zhang X, Qi S, Duan X (2021). Clinical outcomes and safety of polymyxin B in the treatment of carbapenem-resistant Gram-negative bacterial infections: a real-world multicenter study. J Transl Med.

[2] Tacconelli E, Carrara E, Savoldi A (2018). Discovery, research, and development of new antibiotics: the WHO priority list of antibiotic-resistant bacteria and tuberculosis. Lancet Infect Dis.

[3] Bassetti M, Peghin M, Pecori D (2016). The management of multidrug-resistant Enterobacteriaceae. Curr Opin Infect Dis.

[4] CHINET. The bacterial resistance monitoring report of CHINET [EB/OL]. (2023–4–6)[2023–4–6]. http://www.chinets.com/Document/.

[5] Deng Y, Gu JY, Li X (2022). Does Monitoring Total and Free Polymyxin B1 Plasma Concentrations Predict Polymyxin B-Induced Nephrotoxicity? A Retrospective Study in Critically Ill Patients. Infect Dis Ther.

[6] Poirel L, Jayol A, Nordmann P (2017). Polymyxins: Antibacterial Activity, Susceptibility Testing, and Resistance Mechanisms Encoded by Plasmids or Chromosomes. Clin Microbiol Rev.

[7] Sabnis A, Hagart KL, Klöckner A, et al. Colistin kills bacteria by targeting lipopolysaccharide in the cytoplasmic membrane. Elife 2021; 10. 10.7554/eLife.65836 PMC8096433 33821795

[8] Wagenlehner F, Lucenteforte E, Pea F, et al. Systematic review on estimated rates of nephrotoxicity and neurotoxicity in patients treated with polymyxins. Clin Microbiol Infect 2021. 10.1016/j.cmi.2020.12.009 33359542

[9] Lu X, Zhong C, Liu Y (2022). Efficacy and safety of polymyxin E in the treatment of critically ill patients with carbapenem-resistant organism infections. Front Med (Lausanne).

[10] Falagas ME, Kasiakou SK (2006). Toxicity of polymyxins: a systematic review of the evidence from old and recent studies. Crit Care.

[11] Garonzik SM, Li J, Thamlikitkul V (2011). Population pharmacokinetics of colistin methanesulfonate and formed colistin in critically ill patients from a multicenter study provide dosing suggestions for various categories of patients. Antimicrob Agents Chemother.

[12] Falagas ME, Kyriakidou M, Voulgaris GL, Vokos F, Politi S, Kechagias KS (2021). Clinical use of intravenous polymyxin B for the treatment of patients with multidrug-resistant Gram-negative bacterial infections: An evaluation of the current evidence. J Glob Antimicrob Resist.

[13] Hao M, Yang Y, Guo Y, Wu S, Hu F, Qin X. Combination Regimens with Colistin Sulfate versus Colistin Sulfate Monotherapy in the Treatment of Infections Caused by Carbapenem-Resistant Gram-Negative Bacilli. Antibiotics (Basel) 2022; 11. 10.3390/antibiotics11101440 PMC9598655 36290098

[14] Tran TB, Velkov T, Nation RL (2016). Pharmacokinetics/pharmacodynamics of colistin and polymyxin B: are we there yet?. Int J Antimicrob Agents.

[15] Jin J, Zhu J, Zhu Z (2022). Clinical efficacy and nephrotoxicity of intravenous colistin sulfate in the treatment of carbapenem-resistant gram-negative bacterial infections: a retrospective cohort study. Ann Transl Med.

[16] Vardakas KZ, Falagas ME (2017). Colistin versus polymyxin B for the treatment of patients with multidrug-resistant Gram-negative infections: a systematic review and meta-analysis. Int J Antimicrob Agents.

[17] Sisay M, Hagos B, Edessa D, Tadiwos Y, Mekuria AN (2021). Polymyxin-induced nephrotoxicity and its predictors: a systematic review and meta-analysis of studies conducted using RIFLE criteria of acute kidney injury. Pharmacol Res.

